# Lupeol alters viability of SK-RC-45 (Renal cell carcinoma cell line) by modulating its mitochondrial dynamics

**DOI:** 10.1016/j.heliyon.2019.e02107

**Published:** 2019-08-02

**Authors:** Krishnendu Sinha, Sayantani Chowdhury, Sharmistha Banerjee, Bhagirath Mandal, Mullicka Mandal, Sasadhar Majhi, Goutam Brahmachari, Jyotirmoy Ghosh, Parames C. Sil

**Affiliations:** a Department of Zoology, Jhargram Raj College, Jhargram, 721507, West Bengal, India; b Division of Molecular Medicine, Bose Institute, P-1/12 CIT Scheme VII M, Kolkata, 700054, West Bengal, India; c Laboratory of Natural Products & Organic Synthesis, Department of Chemistry, Visva-Bharati University, Santiniketan, 731235, West Bengal, India; d Department of Chemistry, Banwarilal Bhalotia College, Asansol, 713303, West Bengal, India

**Keywords:** Cancer research

## Abstract

Renal cell carcinoma (RCC) is the most common kidney cancer leading to 140,000 deaths per year. Among all RCCs 80% evolve from the epithelial proximal tubular cells within the kidney. There is a high tendency of developing chemoresistance and resistance to radiation therapy in most RCC patients. Therefore, kidney resection is considered as the most effective treatments for patients having localized RCC. There is a high tendency of post-operative recurrence among 20–40% of the patients and this recurrence is not curable. It is also clear that modern medicine has no curative treatment options against metastatic RCC. Lupeol [lup-20(29)-en-3β-ol] is a pentacyclic triterpenoid compound naturally found in various edible fruits and in many traditionally used medicinal plants, and has been demonstrated as effective against highly metastatic melanoma and prostate cancers. The present study was designed to evaluate the effect of lupeol to RCC with molecular details. Treatment with lupeol on SK-RC-45 (a RCC cell line) with the LC_50_ dose of 40μM (for 48 h) induces mitochondrial hyper fission which eventually leads to apoptosis while SK-RC-45 counteracts by enhancing autophagy-mediated selective removal of fragmented mitochondria. This is the first study which concurrently reports the effects of lupeol on RCC and its effect on the mitochondrial dynamics of a cell. Herein, we conclude that lupeol has potential to be an effective agent against RCC with the modulation of mitochondrial dynamics.

## 1 Introduction

**Fig. 1:**
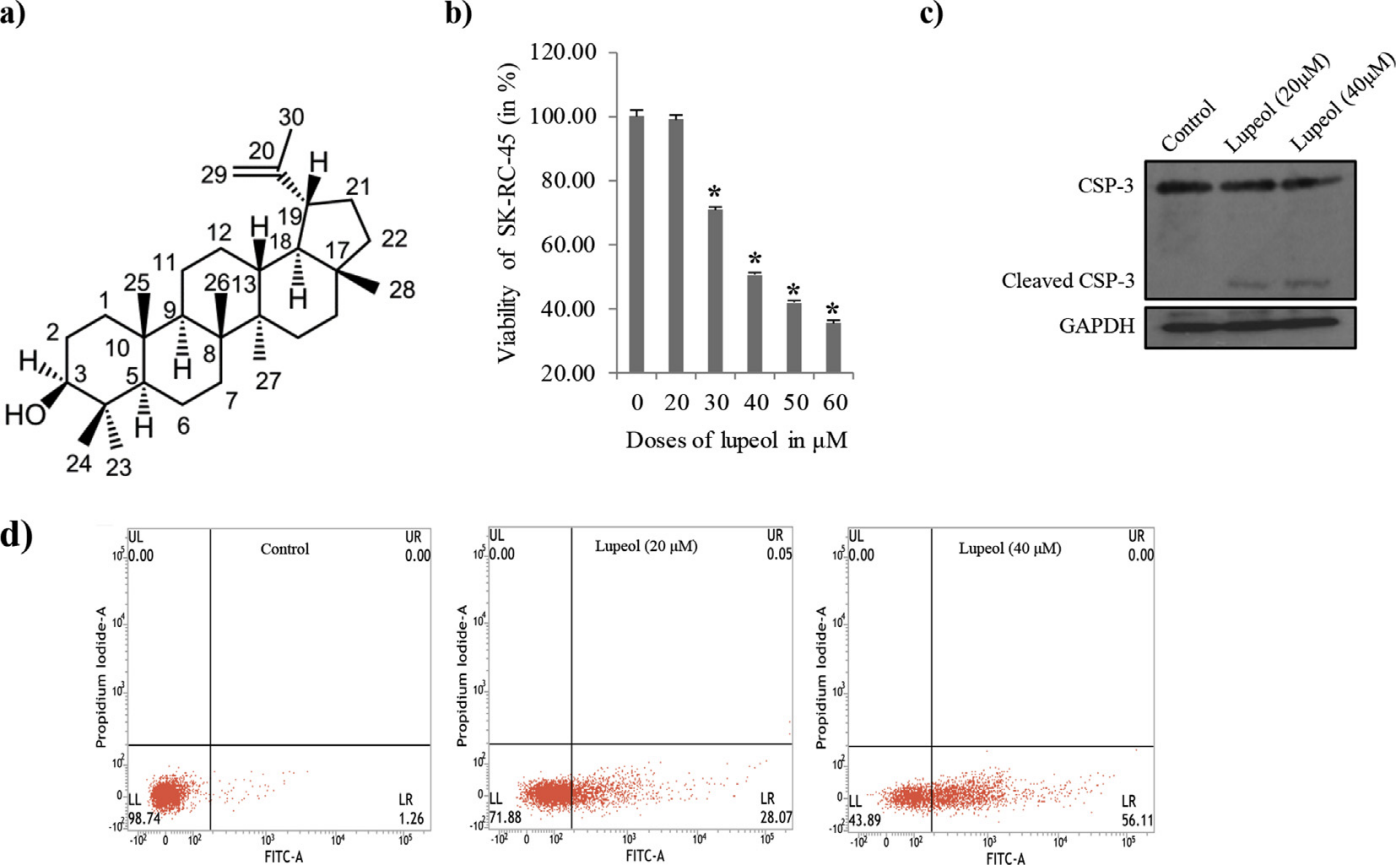
Structure and effect of Lupeol on the viability of SK-RC-45. Control: vehicle treatment alone (for 48 h); Lupeol (20μM): 20μM Lupeol treatment for 48 h; (a) Structure of Lupeol (b) Shows the dose dependent change in viability (expressed in % over control) after Lupeol exposure for 48 h; (c) Immunoblot analysis of Caspase-3 from whole lysate of SK-RC-45 from all three groups. Note that Lupeol activated Caspase-3 (CSP-3) as evident from increased cleaved expression; (d) FACS analysis of Annexin V-FITC staining. Q1_LL: Annexin V negative cells (normal); Q1_LR: Annexin V positive cells (early apoptosis). Values in % in each quadrant represent the amount of cells in that quadrant. ‘FITC-A’ represents FL1-H filter and ‘Propidium iodide A’ represents FL2-H filter. All data are mean ± SEM. P < 0.05 compared with the control group; Data are represented as the mean ± SEM of three individual experiments. *P < 0.05 vs. Control.

Renal cell carcinoma (RCC) is the most common kidney cancer with over 350,000 cases reported annually, causing 140,000 deaths per year [1]. This cancer is reported to be the third leading cause of death for urological tumors [2]. Among various subtypes of RCC, the clear cell tumor subtype accounts for 80% of all RCCs. Epithelial proximal tubular cells are the origin place of this tumor within the kidney, with some instances also representing additional tubular origin [3]. RCC is a pervasive malignancy where one out of five patients having advanced stage already during diagnosis and 30 % of the patients diagnosed with localized tumor develop metastases, even after removal of the parent tumor [3]. Once metastasis is reached, survival rate is 20% up to 5 years and average survival time is nearly one year [4]. Despite of various therapeutic options, proposed outcome of patients with locally advanced tumors or metastatic tumor remains poor [3]. There is a high tendency of developing chemoresistance and resistance to radiation therapy in most RCC patients. Therefore, kidney resection is considered to be the most effective treatments for patients having localized RCC [3]. The postoperative recurrence in RCC patients is around 20%–40% and it is rarely correctable [5]. Thus, there is an obvious necessity to advance treatment options for metastatic RCC [6]. Recently developed remedies targeting vascular endothelial growth factors, platelet growth factors, mammalian target of rapamycin, receptor tyrosine kinases have been reported to show comparatively longer progression-free survival with respect to standard care [7,8], however, the results are still far from being conclusive. For this study we have investigated the effects of lupeol (Fig. 1a) on a RCC cell line, SK-RC-45. We have selected this particular cell line because these cells were generated from a human kidney cancer in the lining of the proximal convoluted tubule (PCT), the most common RCC [9].

The triterpene, lupeol [lup-20(29)-en-3β-ol] (Fig. 1a) is a “secondary metabolite” produced in various edible fruits such as strawberry, grapes, olive, mango, and figs as well as in many vegetables and medicinal plants [10]. Lupeol used in the study has been extracted from the barks of *Bombax ceiba*, an important medicinal plant finding many uses against many diseases manifestations such antimicrobial, wound healing, bone diseases, rheumatoid arthritis, joint pain and many more [11]. Lupeol has been found to possess numerous pharmacological activities (e.g. antioxidant, anti-inflammatory, antimutagenic) both in in vitro and in vivo systems [12]. Researchers have shown that lupeol induces apoptosis in highly metastatic human pancreatic cancer cells such as AsPC-1 [13]. It has also been shown that Lupeol persuades differentiation of mouse melanoma cells [14]. Saleem et al. (2004) reported that Lupeol possesses significant antitumor activity in a two-stage model of mouse skin carcinogenesis [15]. According to their report, Lupeol was found to induce cell cycle arrest and apoptosis in metastatic melanoma cell line 451Lu cells [15].

Mitochondrial dynamics play a crucial role in cell survival and cell death mechanisms. Mitochondrial dynamics refers to the fusion and fusion of mitochondria in the cellular mileau which leads to change of shape and morphology of mitochondria under various physiological as well as pathophysiological conditions. Mitochondrial fission is an well known phenomena in apoptosis The main machineries of fission are dynamin related protein (drp1), mitofusins 1,2 (Mfn 1, 2) and opa 1. The drp1 protein is a GTPase which moves back and forth from cytosol to outer mitochondria membrane of mitochondria under normal condition. Following cellular stress, pro-apoptotic bax and bak is activated. Bax and bak activation leads to sumoylation of drp1 and sumoylated drp1 gets stably attached with mitochondrial membrane. This drp1 assembles in a ring around mitochondria and breaks down the membrane [16]. Mitochondrial function and dynamics are regulated by bcl2 family of proteins. This bcl2 family proteins also influence wide array of cellular and mitochondrial activities like calcium balance, autophagy, mitophagy and bioenergetics [17]. In view of the wide range pharmacologic activities of Lupeol, here, we have shown that Lupeol has significant effect on the survival of SK-RC-45 which is further modulated by the autophagic process of SK-RC-45 cells in in-vitro model. Here, we suggest that Lupeol has the potential to be an effective agent against RCC with the modulation of mitochondrial dynamics [12].

## 2 Materials and methods

### 2.1 Materials and reagents

RPMI-1640 and fetal bovine serum (FBS) were bought from HIMEDIA (Mumbai, India) and HyClone (Thermo Scientific Hy-Clone, Logan, Utah). Methylthiazolyldiphenyl-tetrazolium bromide (MTT) was purchased from Sisco Research Laboratory (Mumbai, India). 3-MA was purchased from Sigma Aldrich (USA). Anti-Beclin-1, anti-LC3B-I/II, anti-Caspase-3 and HRP-tagged secondary antibodies were purchased from Abcam (Cambridge, UK) respectively. Other chemicals used in this study were of highest experimental grade available.

### 2.2 Lupeol isolation

Lupeol was isolated from dried and powdered barks of *Bombax ceiba* as reported by Mukherjee and Roy [18], and verified based on its elemental analyses, physical and detailed spectral properties as described in results section.

### 2.3 Cell culture and treatment

Human Renal Cell Carcinoma (RCC) line, SK-RC-45 were used (obtained as a gift from Dr. Kausik Biswas, Bose Institute) for this study. The obtained cells were maintained in 75-cm^2^ culture flasks at 37 °C in RPMI-1640 medium containing 10% FBS, 100 U/ml penicillin, 100 μg/ml streptomycin, 50 μg/ml gentamicine and 2.5 μg/ml amphotericin B in 5% CO_2_ incubator. For the experimental purpose, Lupeol (filter-sterilized solution) treatment was done at the time of approximately 70% confluency. Lupeol was prepared following the protocol of Mutaza et al. (2009) [13]. Briefly, a stock solution of Lupeol (60 mM) was prepared in warm ethanol (47 °C). Then it was 5 times diluted in DMSO to get a of 12 mM and from it the working dilutions were prepared. The final concentrations of DMSO and alcohol were 0.25% and 0.075% in all the experiments [13]. The experiments were conducted under sterile conditions and the obtained experimental results were determined in triplicate. Lupeol treatment was done in RPMI-1640 media supplemented with 5% FBS.

### 2.4 Treatment of cells

A dose dependent study was carried out where SK-RC-45 cells were treated with 10–60 μM of Lupeol for 48 h along with one only vehicle treated group as control. The media was discarded after 48 h of treatment and the cells were washed twice with phosphate buffered saline (PBS) [19]. The LC_50_ values and optimum time of Lupeol for SK-RC-45 was determined as 40μM and 48h. Cell lysates were prepared and stored at -80 °C for later use.

### 2.5 Study of mitochondrial dynamics

Cells were grown and maintained on sterile glass cover slips following standard procedure [9]. After the incubation period with Lupeol (40μM), the cells were washed in PBS and replaced with pre warmed (37 °C) staining solution (serum free culture medium) containing MitoTracker® Deep Red FM probe at a concentration of 200nM. After incubation and treatment, SK-RC-45 cells were washed and the medium was replaced by the serum-free medium containing 200nM MitoTracker® Deep Red FM and incubated for 30 minutes under appropriate growth conditions. After staining was complete, cells were washed with PBS and fixed and mounted in VECTASHIELD Mounting Medium. All the slides were visualized under Leica TCS SP8. Mitochondrial morphometric parameters were evaluated by Fiji (ImageJ v1.52e) using MiNA macro [20,21]. CLAHE, Median Filter and Tophat preprocessing had been applied to data images in MiNA analysis. These tools, and documentation regarding their usage, are available at https://github.com/ScienceToolkit/MiNA.The FIJI distribution of ImageJ on which the macros are designed to run is available at https://fiji.sc/.

### 2.6 Effects of autophagy inhibitors on cell viability

The cell viability was assessed with MTT assay. In briefly, 1×10^5^ cells were seeded into each well in 96-well culture plates. SK-RC-45 cells were treated with medium containing vehicle only (Control), 40μM of Lupeol, and 3-MA at a concentration of 5 mM respectively for 48 h. 3-MA was added to the medium 1 h prior to the addition of Lupeol. After incubation the cell viability was assessed by MTT assay described by Sinha et al. (2014) [9].

### 2.7 Cell lysis and immunoblotting

Cells were lysed with RIPA buffer supplemented with protease and phosphatase inhibitor. After addition of RIPA buffer, cells were vigorously vortexed and subjected to repeated freeze-thaw cycle followed by brief sonication. The lysates were centrifuged at 12,000 rpm at 4 °C for 10 min. The supernatant was collected and protein content was measured using Bradford reagent. For Western blot analysis, an equal amount of protein (50 μg) from respective experimental group was subjected to 10–12% SDS-PAGE and transferred to PVDF membranes. Nonspecific binding on the membrane was prevented by keeping the membrane in blocking buffer containing 5% BSA for 2 hours at room temperature. After blocking, the membranes were incubated with anti-Beclin-1 (1:1000 dilution) primary antibody at 4 °C overnight. The membranes were washed in TBST for 30 min followed by incubation with appropriate HRP-conjugated secondary antibody (1:15,000 dilution) for 2h at room temperature. Expression of proteins were detected using HRP substrate ECL solution.

### 2.8 Lupeol treatment on si-RNA mediated Bcl-2 knockdown cells

In brief, 1 day before transfection, 1.5 × 10^5^ cells were plated in 6 well plates in antibiotic free complete RPMI-1640. Transfection was done by 150 picomole siRNA (Bcl-2 or scramble) using 5μl of Lipofectamine-2000. 12 h post-transfection, 5 × 10^4^ cells were plated in a 12-well plate. These transfected cells were subjected to Lupeol treatment (i.e. 40 μM for 48 h). After treatment the viability was measured using MTT assay [19,22].

### 2.9 RNA extraction and reverse transcriptase PCR

RNA extraction and RT PCR was performed to confirm the silencing of bcl2 gene expression by si RNA treatment. RNA was extracted from cells with the help of TRIZOL reagent. Quanitity of RNA was measured specrtophometrically with the help of nanodrop HellmaTrayCell Type 105.810 (Hellma Analytical). One microgram of RNA was converted to cDNA with the help of Thermo Scientific Verso cDNA synthesis kit (Thermo Scientific, USA).

The thermal cycling was as follows: 95 °C for 5 min (initial denaturation) followed by the set of 35 cycles: 95 °C for 30 sec (denaturation), 55 °C for 30 sec (primer annealing), and 72 °C for 45 sec (primer extension). DNA extension step was done at 72 °C for 5 min. The PCR products were held at 4 °C. The PCR products were resolved at 1.5% agarose gel. The details of the primer are: Bcl2 Forward: 5′GAGCACAGAAGATGGGAACA 3′ Reverse: “CCACTGTCACTCTTGCAAATTC”. GAPDH Forward “CGACCACTTTGTCAAGCTCA” Reverse: “TTCCTCTTGTGCTCTTGCTG”

### 2.10 Detection of cell death by annexin V affinity assay

SK-RC-45 cells were treated as per described in the method section previously. The cells were scraped and centrifuged at 300g for 5 min at room temperature at the end of respective incubation time (Sinha et al., 2014). The pellets were collected and washed with PBS. After PBS wash, in 1X Annexin V Binding Buffer was used to resuspend the pellets followed by incubation for 5 mins in dark with 1μl of Annexin V/at RT. The samples were then analyzed at FACS Verse with an excitation at 488 nm and emission at 520 nm respectively using the FACSuite software.

### 2.11 Statistical analysis

Results are expressed as mean (±SEM) from three independent experiments. Statistical evaluation were done with the help of means of one-way analysis of variance (ANOVA) and the group means were compared through Tukey test. p-value less than 0.05 were considered as statistically significant.

## 3 Results

### 3.1 Isolation of lupeol

Isolated Lupeol was verified based on its elemental analyses, physical and spectral properties as described: white microcrystalline powder; mp 205–207 °C; IR (KBr): ν_max_ = 3325 (OH), 3068, 1637, 1032, 881 cm^−1^. ^1^H NMR (400 MHz, CDCl_3_): δ = 0.69-0.67 (m, 1 H, H-5), 0.76 (s, 3 H, H-23), 0.78 (s, 3 H, H-24), 0.83 (s, 3 H, H-25), 0.90-0.86 (m, 1 H, H-1a), 0.94 (s, 3 H, H-26), 0.96 (s, 3 H, H-27), 1.03 (s, 3 H, H-28), 1.11-1.05 (m, 1 H, H-12b), 1.23-1.16 (m, 2 H, H-22), 1.26-1.25 (m, 2 H, H-21), 1.29-1.28 (m, 1 H, H-11b), 1.33-1.31 (m, 1 H, H-9), 1.36-1.35 (m, 1 H, H-1b), 1.39-1.37 (m, 1 H, H-11a), 1.39-1.37 (m, 1 H, H-18), 1.39-1.37 (m, 2 H, H-6), 1.47-1.40 (m, 2 H, H-7), 1.57-1.48 (m, 2 H, H-16), 1.60-1.58 (m, 2 H, H-15), 1.60-1.58 (m, 1 H, H-13), 1.65-1.62 (m, 2 H, H-2), 1.68 (s, 3 H, H-30), 2.00-1.88 (m, 1 H, H-12a), 2.41-2.34 (m, 1 H, H-19), 3.19-3.17 (m, 1 H, H-3), 4.57-4.56 (m, 1 H, H-29b), 4.68 (d, *J* = 2.4 Hz, 1 H, H-29a) ppm. ^13^C NMR (100 MHz, CDCl_3_): δ = 14.69 (C-27), 15.51 (C-24), 16.12 (C-26), 16.27 (C-25), 18.15 (C-28), 18.47 (C-6), 19.45 (C-30), 21.08 (C-11), 25.30 (C-12), 27.56 (C-15), 27.60 (C-23), 28.14 (C-2), 30.00 (C-21), 34.44 (C-16), 35.74 (C-7), 37.32 (C-10), 38.20 (C-13), 38.86 (C-1), 39.01 (C-4), 40.15 (C-22), 40.99 (C-8), 42.98 (C-17), 43.15 (C-14), 48.13 (C-18), 48.45 (C-19), 50.59 (C-9), 55.45 (C-5), 79.16 (C-3), 109.46 (C-29), 151.12 (C-20) ppm. HRESIMS: *m/z* 449.3751 [M + Na]^+^ (Cald. for C_30_H_50_ONa at *m/z* 449.3759). Elemental analysis: calcd (%) for C_30_H_50_O: C, 84.44; H, 11.81; Found: C, 84.48; H, 11.79.

### 3.2 Dose and time dependent effect of lupeol on SK-RC-45

Fig. 1b shows the dose dependent effect of Lupeol on SK-RC-45. We observed an approximately two fold decrease in cell viability upon 40μM Lupeol treatment over 48 h . Hence, the dose of 40μM for 48 h was found to be the LC_50_ of Lupeol for SK-RC-45. We used this LD50 dose and a lower less effective dose (20μM) for further experimentations to observe progressive dose dependent changes. We also found a significant increase over control in caspase-3 cleavage at these doses [Fig. 1c, Fig S1(c) supplementary figure]. Apoptosis was also confirmed through annexin-V/FITC binding assay with the help of flowcytometry (Fig. 1d).

### 3.3 Lupeol induces mitochondrial fission in SK-RC-45

**Fig. 2:**
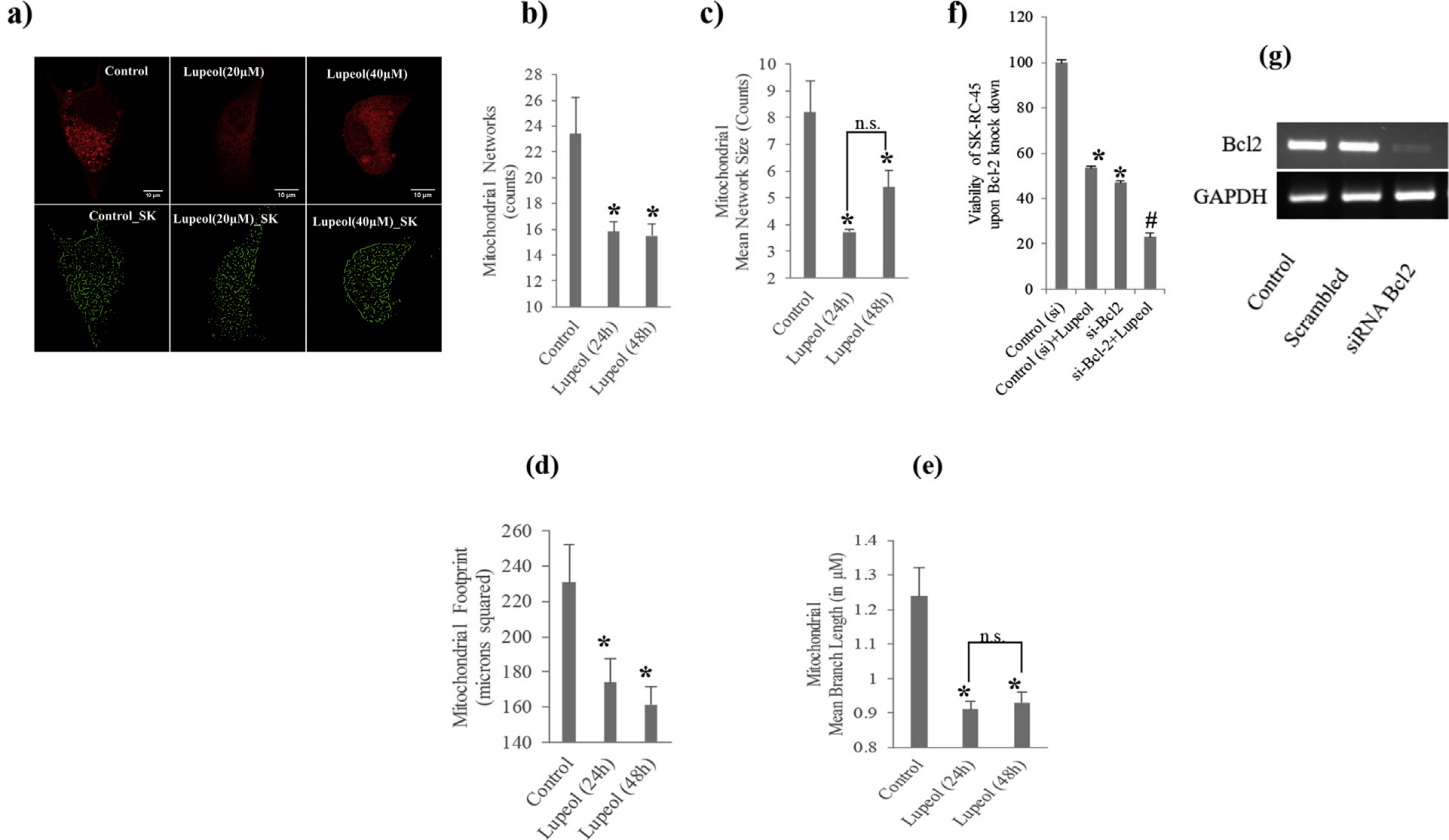
Effect of Lupeol on the mitochondrial dynamics of SK-RC-45 and its relation with Bcl-2. Control: vehicle treatment alone (for 48 h); Lupeol (20μM): 20μM Lupeol treatment for 48 h; Lupeol (40μM): 40μM Lupeol treatment for 48 h; Control_SK: Skeletonized version of Control from MiNA analysis; Lupeol (20μM): Skeletonized version of Lupeol (20μM) from MiNA analysis; Lupeol (40μM): Skeletonized version of Lupeol (40μM) from MiNA analysis (see the ‘Materials and methods’ for MiNA). (a) Confocal image and respective skeletonized version of representative SK-RC-45. Results of MiNA analysis (b) Networks (this is the number of objects in the image that contain at least 1 junction pixel and are thus comprised of more than one branch), (c) Mean Network Length (The average length of all rods/branches), (d) Mean Network Size (This is the mean number of branches per Network) and (e) Mitochondrial Footprint (This is the total area in the image consumed by signal after being separated from the background that is proportionate with the actual mitochondrial mass). (f) Effect of Bcl-2 knock down on Lupeol's efficacy. (g) RT PCR of Bcl-2 to confirm the knockdown of Bcl-2 m RNA after treatment of cells with Bcl-2 si RNA. Control (si): Control scramble si-RNA treated cells; Control (si)+Lupeol: Control scramble si-RNA treated cells with 40μM Lupeol treatment for 48 h; si-Bcl-2: Bcl-2 si-RNA treated cells; si-Bcl-2+Lupeol: Bcl-2 si-RNA treated cells with 40μM Lupeol treatment for 48 h control. Data are represented as the mean ± SEM of three individual experiments. *P < 0.05 vs. Control. #P < 0.05 vs. Lupeol n. s. indicates ‘non-significant’ difference.

Fig. 2a shows the architecture of mitochondrial network and status of mitochondrial dynamics at the specific time point. For this purpose we extract four vital information from the images namely Networks, Mean Length, Mean Network Size and Mitochondrial Footprint (Fig. 2). We observed dose dependent significant decrease of all these four parameters in Lupeol treated cells, in respect to control (Fig. 2b-e). In this respect we were also interested to investigate the effect of Bcl-2 in the whole process as we know that this anti-apoptotic protein have significant effect on the mitochondrial dynamics. Fig. 2f shows that the Bcl-2 knock down significantly increased the efficacy of Lupeol's effect on SK-RC-45 Fig. 2g [Fig S2(g); supplementary figure], RT PCR data confirms the knockdown of Bcl2 gene using siRNA in SKRC45 cell line.

### 3.4 SK-RC-45 alienates the effect of lupeol by inducing autophagy

**Fig. 3:**
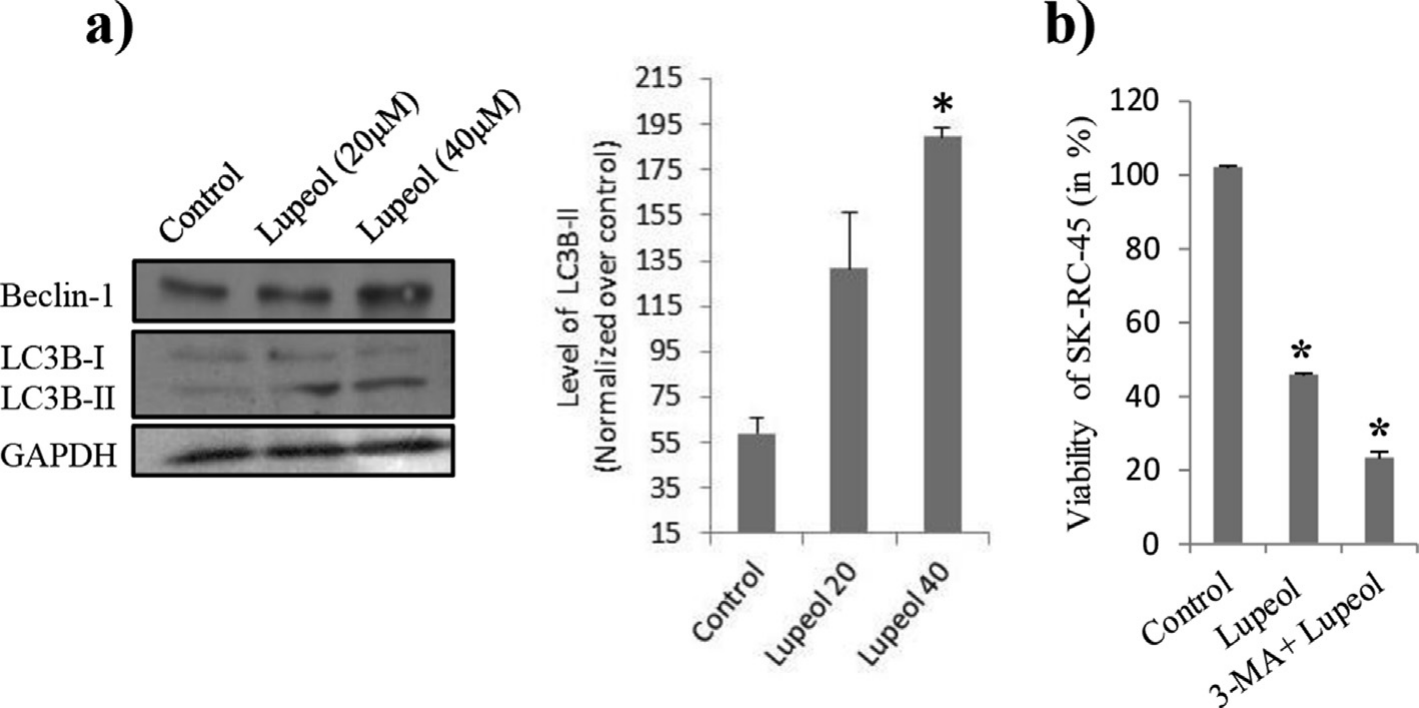
SK-RC-45's response in respect to Lupeol treatment. Control: vehicle treatment alone (for 48 h); Lupeol (20μM): 20μM Lupeol treatment for 48 h; Lupeol (40μM): 40μM Lupeol treatment for 48 h. (a) Immunoblot analysis of Beclin-1and LC-3B I/II from whole lysate of SK-RC-45 from all three groups. Note that, upon Lupeol treatment cellular Beclin-1 and LC-3B II level (thus promotes LC-3B I to LC-3B II conversion) increased. (b) Effect of 3-MA pretreatment on Lupeol treatment. Note that, upon 3-MA pre-treatment cell death significantly induced upon Lupeol treatment in respect to Lupeol treatment alone. Here Lupeol was used at a dose of 40 μM for 48 h *P < 0.05 vs. Control.

[Fig. 3a, Fig S3 (a) supplementary figure] shows the status of autophagy in Lupeol treated SK-RC-45 cells. We observed significant increase of autophagic cellular markers, Beclin-1 and LC-3B II in a dose dependent manner. We also found that, over the same experimental conditions, cellular survivality significantly decreased upon inhibition of autophagy (Fig. 3b). This signifies that autophagy was initiated by cells (not by Lupeol) in response to Lupeol as a protective action.

## 4 Discussion

In this study, we have established that (1) the administration of Lupeol significantly reduces the viability of RCC cell SK-RC-45; (2) it alters the mitochondrial dynamics by inducing mitochondrial fission which sequentially leads to apoptosis; (3) cell alienates the process by inducing autophagy response probably with an aim to remove fragmented mitochondrial copies.

The triterpene, Lupeol has already been found to possess anticancer effect and tested over several types of cancers. But its effect on RCC was unknown. Here in this study we have found that Lupeol have profound effect on the viability of the RCC cell, SK-RC-45 (Fig. 1). Mitochondria is an extremely dynamic organelle [23,24]. It is in an equilibrated process of continuous fusion and fission [25]. Fusion formed extensive network structure whereas under fission network structure shortens [16]. In general, under stressful condition the dynamics tilted towards fission which leads to mitophagy which decreases mitochondrial mass and thus cell tries to recover from the stress [26]. But overwhelming stress leads to hyper-fission of mitochondria which ultimately leads to extensive loss of mitochondrial mass. In such situation cells cannot survive further and consequently enters apoptosis [26]. In our study we found that Lupeol imparts its effect on SK-RC-45 by tilting the dynamics towards fission. It is evident from the result (Fig. 2). Upon treatment with Lupeol, total number of mitochondrial networks got significantly reduced (Fig. 2b). This result indicates that the fission produces punctate or rod shaped mitochondria and thus reduces the network structure. This result is confirmed by other two parameters called mean mitochondrial network size and mean branch length. This results show that upon Lupeol treatment both the number of branches per network and mean length of the branches which were resulted from excessive mitochondrial fission. Mitochondrial footprint was also reduced by Lupeol treatment, possibly relating to possibly relating to mitophagy of the fragmented mitochondria [27].

After confirming Lupeol's effect on the SK-RC-45 we would like to see the status of autophagy in the cell upon Lupeol treatment as our study indicates extensive mitochondrial clearing which is mainly done by autophagy. In our quest, we found that autophagy was induced upon Lupeol treatment (Fig. 3). We have found increased level of Beclin-1 and increased LC-3BII conversion both of which are potent markers of autophagy. Then we would like to investigate the background reason for such an autophagic flux. It could be induced by Lupeol itself or it could be a protective response of cell to overcome the effect of Lupeol, more specifically for removing fragmented mitochondria. Hence we block the autophagic flux by 3-MA (a potent autophagy blocker). We found that blocking autophagy significantly lowered cell survivability upon Lupeol treatment (Fig. 3b). This signifies the protective effect of autophagy upon Lupeol treatment.

It is well known that Bcl-2 have a pro-survival anti-apoptotic role. On mitochondrial outer membrane it also form heterodimer with Bax, a pro-apoptotic protein and a prominent mitochondrial fission inducer [16,28,29,30]. So it can be easily postulated that in the absence of Bcl-2 would augment the mitochondrial fission and cell death. From our study we also found the same result. We found that Bcl-2 knock down augment the effect of Lupeol thus, mitochondrial fission and cell death (Fig. 2f).

Therefore, the outcome of the study indicates that, Lupeol induces mitochondrial hyper fission which ultimately leads to apoptosis. Cell tries to protect itself by augmenting autophagy mediated selective removal of fragmented mitochondria which is also evident from the loss of mitochondrial footprint upon Lupeol exposure. The study is one of its kind as it is the first report showing the excellent pharmacological effects of Lupeol against the RCC and also the first to report the effect of Lupeol on the mitochondrial dynamics of a cell.

## Declarations

### Author contribution statement

Parames C. Sil, Goutam Brahmachari: Conceived and designed the experiments; Analyzed and interpreted the data; Contributed reagents, materials, analysis tools or data; Wrote the paper.

Krishnendu Sinha, Sayantani Chowdhury, Sharmistha Banerjee: Conceived and designed the experiments; Performed the experiments; Analyzed and interpreted the data.

Mullicka Mandal: Performed the experiments; Wrote the paper.

Bhagirath Mandal, Sasadhar Majhi: Performed the experiments; Contributed reagents, materials, analysis tools or data, Wrote the paper.

Jyotirmoy Ghosh: Conceived and designed the experiments; Analyzed and interpreted the data; Wrote the paper.

### Funding statement

This work was supported by the Council of Scientific & Industrial Research, New Delhi who provided G. Brahmachari with a financial grant [No. 02(0260)/2016/EMR-II], and the University Grant Commission Minor Research Project who provided financial support to Jyotirmoy Ghosh [No. F.PSW-014/15-16(ERO)].

### Competing interest statement

The authors declare no conflict of interest.

### Additional information

No additional information is available for this paper.

## Appendix A Supplementary data

Sinha et al Supplementary HELIYON_2018_6045 R2 Sinha et al Supplementary HELIYON_2018_6045 R2
