# Supplementary material for: Lupeol alters viability of SK-RC-45 (Renal cell carcinoma cell line) by modulating its mitochondrial dynamics
Source: Heliyon. 2019 Aug 2;5(8):e02107. doi: 10.1016/j.heliyon.2019.e02107 (PMC6690575; doi:10.1016/j.heliyon.2019.e02107)
Supplement: Sinha et al Supplementary HELIYON_2018_6045 R2 [file mmc1.docx]

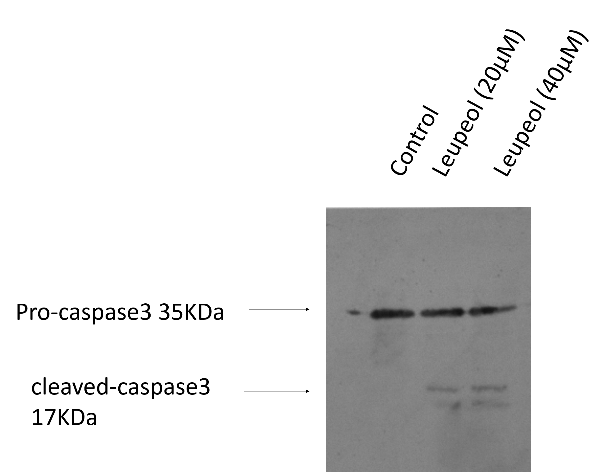


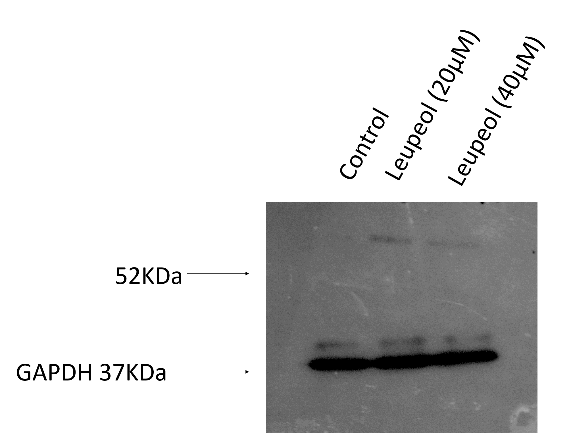


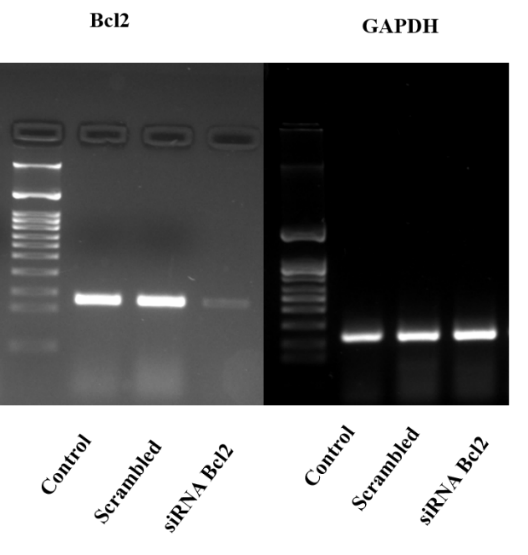
FigureS1(c) Immunoblot analysis of Caspase-3 from whole lysate of SK-RC-45 from all three groups. Note that Lupeol activated Caspase-3 (CSP-3) as evident from increased cleaved expression.

FigureS2(g) RT PCR of Bcl-2 to confirm the knockdown of bcl-2 m RNA after treatment of cells with Bcl-2 si RNA. (h) densitometry of RT PCR analysis of Bcl-2


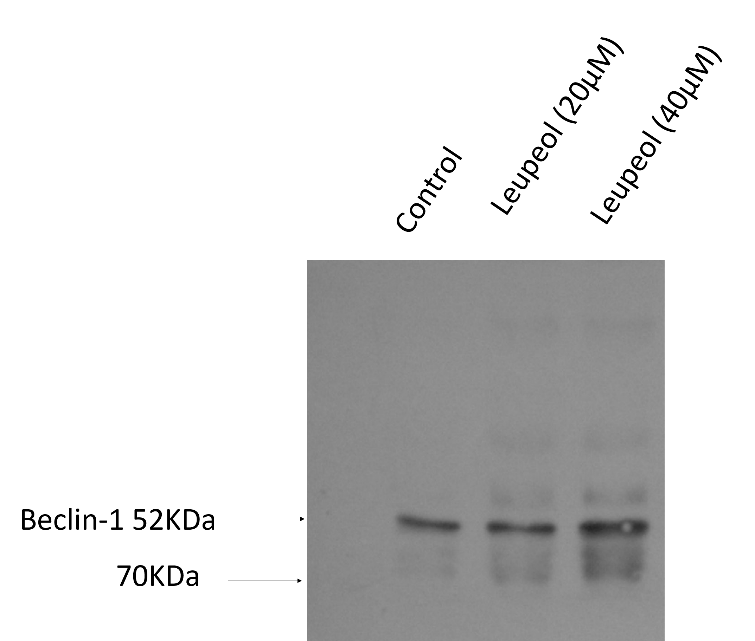


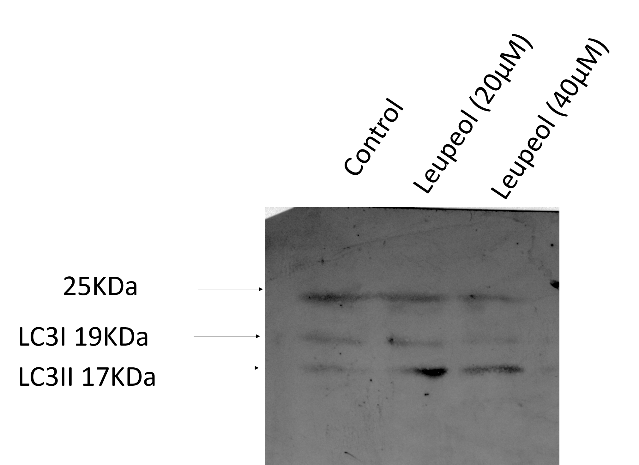


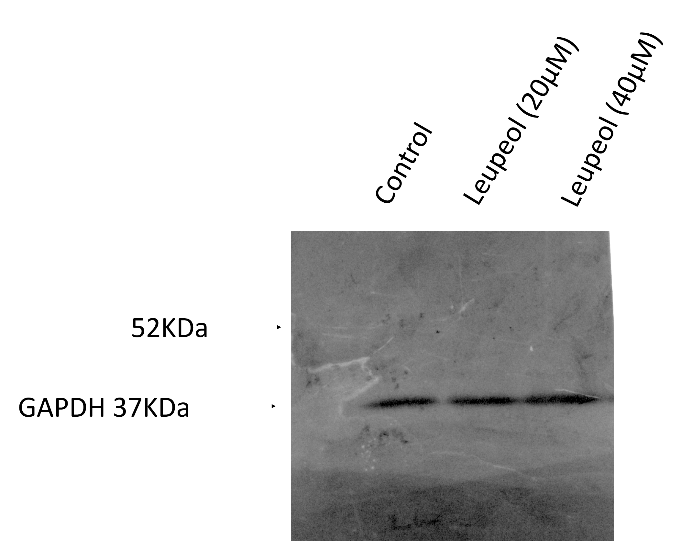


FigureS3 (a) Immunoblot analysis of Beclin-1and LC-3B I/II from whole lysate of SK-RC-45 from all three groups. Note that, upon Lupeol treatment cellular Beclin-1 and LC-3B II level (thus promotes LC-3B I to LC-3B II conversion) increased.
